# Visual Field Tests: A Narrative Review of Different Perimetric Methods

**DOI:** 10.3390/jcm13092458

**Published:** 2024-04-23

**Authors:** Bhim Bahadur Rai, Faran Sabeti, Corinne Frances Carle, Ted Maddess

**Affiliations:** 1 John Curtin School of Medical Research, Australian National University, Canberra, ACT 2601, Australia; faran.sabeti@canberra.edu.au (F.S.); corinne.carle@anu.edu.au (C.F.C.); ted.maddess@anu.edu.au (T.M.); 2 Faculty of Health, School of Optometry, University of Canberra, Canberra, ACT 2601, Australia

**Keywords:** Amsler grid, objective perimetry, perimetric methods, principle of redundancy, visual field tests

## Abstract

Visual field (VF) testing dates back to fifth century B.C. It plays a pivotal role in the diagnosis, management, and prognosis of retinal and neurological diseases. This review summarizes each of the different VF tests and perimetric methods, including the advantages and disadvantages and adherence to the desired standard diagnostic criteria. The review targets beginners and eye care professionals and includes history and evolution, qualitative and quantitative tests, and subjective and objective perimetric methods. VF testing methods have evolved in terms of technique, precision, user-friendliness, and accuracy. Consequently, some earlier perimetric techniques, often still effective, are not used or have been forgotten. Newer technologies may not always be advantageous because of higher costs, and they may not achieve the desired sensitivity and specificity. VF testing is most often used in glaucoma and neurological diseases, but new objective methods that also measure response latencies are emerging for the management of retinal diseases. Given the varied perimetric methods available, clinicians are advised to select appropriate methods to suit their needs and target disease and to decide on applying simple vs. complex tests or between using subjective and objective methods. Newer, rapid, non-contact, objective methods may provide improved patient satisfaction and allow for the testing of children and the infirm.

## 1. Introduction

Accurately tested visual fields (VFs) provide clues to several ophthalmological and neurological diseases. The VF test is the most commonly performed clinical diagnostic test for the evaluation of extrafoveal visual function for the diagnosis and monitoring of neuro-retinal diseases [1]. It is an integral part of a comprehensive ophthalmic evaluation.

The objective of this narrative review is to bring all the perimetric methods together for easy access to beginners and eye care professionals. It encompasses the history and evolution of VF test methods, including different subjective perimetric methods, standard automated perimetry (SAP), and objective perimetric methods. We discuss the advantages, disadvantages, and ability to meet desired diagnostic standards of different methods so clinicians are able to make the right choice when choosing perimetric methods.

## 2. Materials and Methods

We researched online for different VF testing and perimetric methods. We have mainly used the Web of Science (WoS, Clarivate, London, UK) as the search tool. The keywords or terms used to search for relevant literature were visual field tests, perimetric methods, standard automated perimeter, subjective perimetric methods, objective perimetric methods, and principles of perimetry.

## 3. Relevant Sections

We included the findings of our review of the literature under the following subheadings.

### 3.1. History and Evolution of Perimetry

Historically, peripheral VF evaluation was first performed by Hippocrates in fifth century B.C., when he reported a hemianopia [2,3]. Ptolemy attempted to quantify the VF and described it as roughly circular in 150 B. C., and the first recorded extramacular VF testing was accomplished by Galen in 175 A.D. In 1602, Ulmas published the first VF illustration. Mariotte defined the physiological blind spot, relating it to the optic disc in 1668 [2]. By the early 19th century, Thomas Young had measured the extent of normal VF to be 50°, 60°, 70°, and 90° superiorly, nasally, inferiorly, and temporally, respectively. Less than a century later, Albrecht von Graefe pioneered the applications of perimetry and VF testing in clinical ophthalmology [3]. 

**Figure 1:**
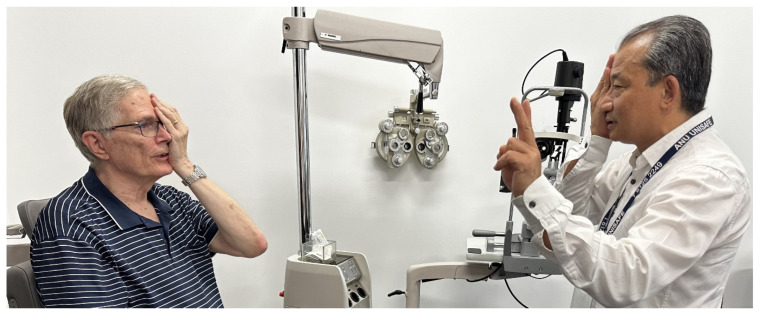
Confrontation test. The patient faces the examiner at a 1 m distance with eyes at the same level as the examiner’s. Each fixates their vision on the other’s opposite eye while covering their contralateral eye with the palm of the hand (avoiding pressure on the eye). The examiner moves a target object from the periphery towards the midline in all four quadrants: superonasal, superotemporal, inferonasal, and inferotemporal field of vision. Alternatively, the examiner can test each quadrant in the patient’s VF by having them count the number of fingers that they are showing. The patient indicates when the target is visible. Photo courtesy of Corinne Carle. In the photo, Ted Maddess is a patient and Bhim Rai is the examiner.

VF testing methods are categorized into qualitative or quantitative. Qualitative methods were used early on when the technique was in its beginning stage. The confrontation test was the most practiced method. The examiner and patient face each other approximately a meter apart, looking into each other’s eyes. Both cover one eye with the palm of their hand. The examiner slowly moves their finger, or a target object, from the far periphery to the central field midway between them, asking the patient to indicate when the target is detected. The procedure is repeated for the four quadrants or in a different meridian if required and with another eye. Alternatively, the examiner can test each quadrant in the patient’s VF by having them count the number of fingers that they are showing (Figure 1) [4]. Assuming that the examiner has a normal VF, the findings of the patient are then compared with those of the examiner [3]. 

**Figure 2:**
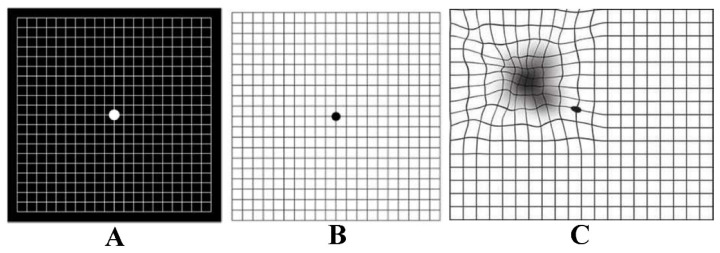
Amsler grid test. (**A**) Original Chart 1 Amsler grid with normal report [8]; (**B**) modified Amsler grid with normal report; (**C**) report showing visual field defect—distortion and scotoma. Reprinted from Kanski’s Clinical Ophthalmology, 8th Edition (2015) by Brad Bowling, with permission from Elsevier.

Another qualitative VF method is the Amsler Grid. It is a basic test for the central VF, performed by placing a printed grid at a reading distance (30–33 cm), and the patient reports any missing or distorted part of the grid with each eye consecutively [5]. The technique has good efficacy for diagnosing central VF defects [6], and is commonly used to monitor macular degeneration. There are different types of grids: the original Chart 1 has white lines on a black background (Figure 2). The outer grid encloses 400 smaller 5 mm squares, each small square subtending an angle of 1° when viewed at 1/3 of a meter. The modified version of Chart 1 has black lines on a white background [7]. The test results are graded as normal, distorted grid part (metamorphoma), or missing grid portion (scotoma) [7]. 

**Figure 3:**
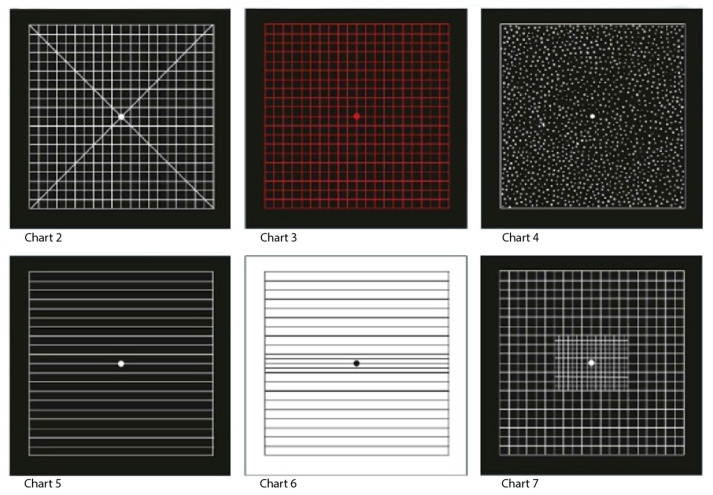
Different types of Amsler grids. Chart 2 has two diagonals to help fixation for patients with a central scotoma. Chart 3 has a red-on-black background to stimulate long wavelength foveal cones and is useful in detecting subtle colour scotomata in toxic maculopathy, optic neuropathy and chiasmal lesions. Chart 4 consists of only random dots and is used to distinguish scotoma from metamorphopsia, as there is no form to be distorted. Chart 5 consists of horizontal lines and is designed to detect metamorphopsia along specific meridians for patients with reading difficulty. Chart 6 is applied for more fine evaluation. Chart 7, in addition, has a fine central grid with each angle subtending half a degree, so it is more sensitive [8]. Reprinted from Kanski’s Clinical Ophthalmology, 8th Edition (2015) by Brad Bowling, with permission from Elsevier.

To suit different patient profiles, there are six other types of Amsler grids, charts 2–7, as shown in Figure 3 [8].

**Figure 4:**
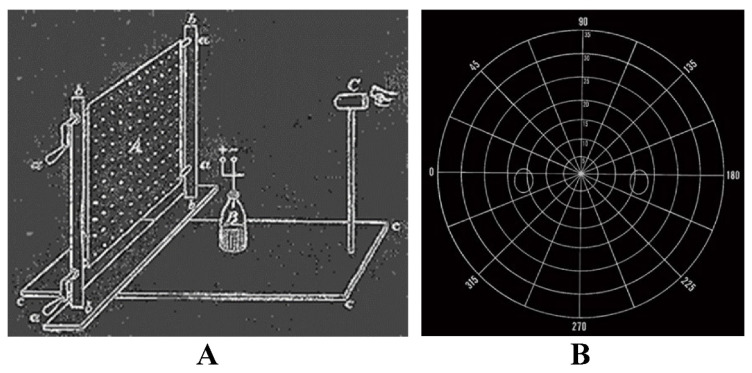
Tangent screen. (**A**) Original Tangent screen [10], (**B**) Bjerrum’s Tangent screen. Reprinted from measurement of the visual field limits: the perimeter, with permission from the Imaging and Perimetry Society.

Tangent screen technique, also known as Bjerrum’s screen, is a quantitative method initially popularized by Jannik Bjerrum and further developed by Harry Traquair [9]. The screen is a flat, usually black surface, used for detailed mapping of central 30° of VF (Figure 4). It is made of black matte material and stitched with radial lines at 15° intervals and circles at 5° intervals. It measures 64″ high × 44″ wide and is used at 1 m with Traquair or similar stimuli. 

**Figure 5:**
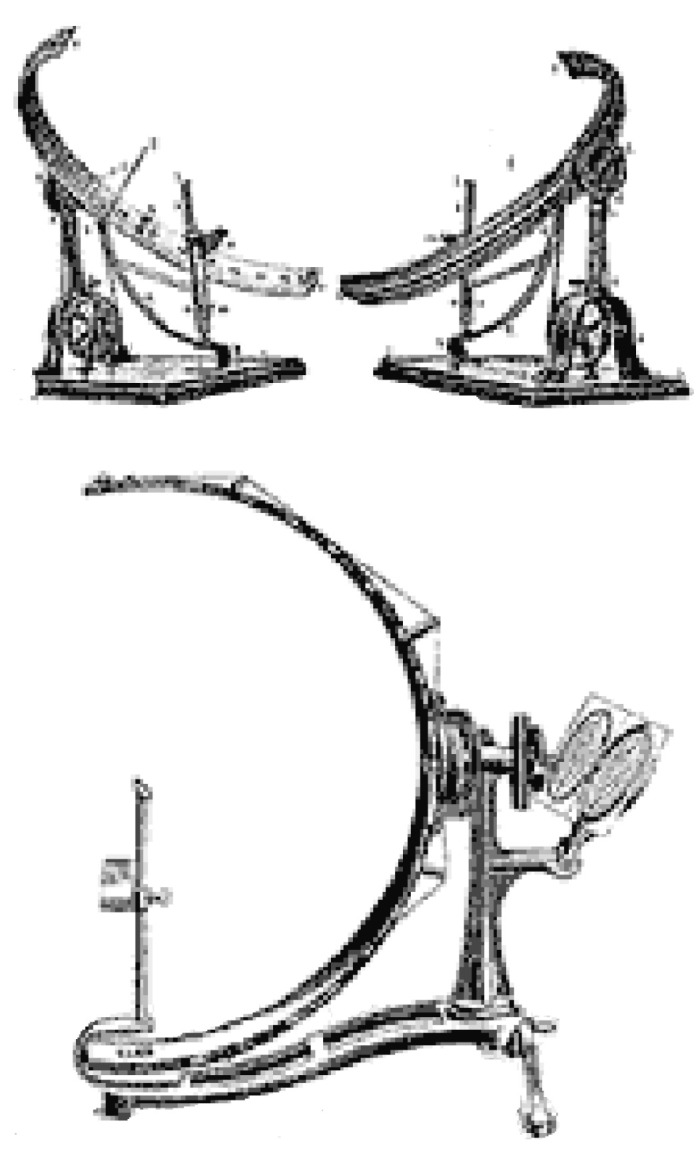
Three views of the Förster Arc Perimeter [11]. Reprinted from measurement of the visual field limits: the perimeter, with permission from the Imaging and Perimetry Society.

The main limitation of the Bjerrum Tangent Screen is that it only measures the central 30° of the VF [3]. To overcome this limitation and provide a method of testing full peripheral vision, with stimuli at a constant distance from the eye, Hermann Rudloph Aubert (psychiatrist) and Carl Friedrich Richard Förster (ophthalmologist) invented the Arc Perimeter in 1869 (Figure 5), which is commonly known as the Förster Perimeter [3]. 

The Arc Perimeter evaluated the full extent of peripheral VF but was again limited by inconsistent background illumination and therefore adaptation. In 1945, Hans Goldmann brought enormous improvement to modern perimetry by developing a hemispheric bowl perimeter with uniform background illumination capable of performing both static and kinetic perimetry using varied targets in terms of size, luminance, and colour. Acknowledging his noble work, he was honoured by this perimeter being named the Goldmann Perimeter [12]. 

### 3.2. The Age of Automation: Goldmann Bowl Perimetry

Disadvantages of manual perimetry included inaccurate results stemming from errors by both patients and examiners. This led to the invention of automated perimetry [13]. Among many investigators, Franz Fankhauser and his co-workers were undoubtedly the forerunners in developing Octopus, the first automated perimeter [14]. He was honoured as the Father of the Automated Perimeter [15]. Anders Heijl and his colleagues developed the Humphrey Field Analyzer, instituting different types of VF tests and analytical methods within one device [16]. With the availability of new computer-controlled technology, automated static perimeters were developed. Equipment made by Octopus and later by Humphrey became nearly universal as the dominant VF tools [17]. Goldmann kinetic perimetry originally used various dot sizes of stimuli to define isopters. Goldmann stimulus size III (henceforth GSIII), with its 0.43° diameter and 4.0 mm^2^ area, is commonly used; size V, with 1.72° diameter and 64 mm^2^, is used for patients with poor visual acuity, while sizes I, II, and IV are rarely used clinically. Since the GSIII was deemed as being the most useful of the set, both the Octopus and Humphrey settled on using the GSIII because, for all SAP, the use of a single stimulus size was recommended [18]. In kinetic perimetry, when the stimulus traverses the field, it can paint much of the field, so the area tested is greater, and nearly complete coverage can be obtained. In static perimetry, the coverage is low. In 2007, the production of the Goldmann perimeter ceased and was replaced by the Octopus 900 perimeter (Haag Streit International, Koeniz, Switzerland). The Octopus 900 has a kinetic perimetry program similar to the Goldmann kinetic perimetry, and the results are comparable [19]. 

## 4. Subjective Perimetric Methods

### 4.1. Standard Automated Perimetry (SAP)

Automated perimetry is the cornerstone for the assessment of retinal functional loss and detect its progression [20]. The strategies of the Goldmann Perimeter developed in 1945 are still applied today in SAP. It uses white stimuli against a dimmer white background for adaptation to a standard background level to assess the threshold at which a GSIII can be detected. In static perimetry, each stimulus of varying luminance is presented at predetermined locations until it is recognized by the patient. In kinetic perimetry, a stimulus of fixed luminance is moved from the periphery towards the central VF until it is visible to the patient with their vision fixated in the centre [18]. For many years, different test strategies and perimeters have been developed and applied in clinics. The development of the Swedish Interactive Threshold Algorithm (SITA) strategy and Guided Progression Analysis (GPA) has perhaps made SAP the method of choice [20]. SAP is performed using one of four tests: 30-2 or 24-2 SITA Standard^TM^ or 30-2 or 24-2 SITA Fast^TM^. SITA Standard provides relatively accurate reports and has a test time of about 4 to 8 min per eye, depending on the test point pattern used and the degree of VF loss. SITA Fast takes only 2 to 6 min per eye with diagnostic sensitivity equal to that of a full threshold test [21]. A new SITA perimetric threshold testing algorithm called the SITA Faster has recently been introduced on the Humphrey Field Analyser (HFA) 3 device. Its test time is 30% shorter than SITA Fast and 53.5% shorter than SITA Standard at comparable mean deviation [22,23,24]. There is also a 30-2 version of SITA Faster, but it still takes 6 min to test an eye [25].

### 4.2. Redundancy Limits SAP

The detection of visual stimuli needs an intact neural pathway. VF loss is the result of damage to all three types of RGCs, namely parvocellular (P), magnocellular (M), and koniocellular (K). Although the transmission of light signals to the brain is the function of all RGCs, the subtypes have different functions. P cells transmit information about red/green colour and form and have high spatial resolution. M cells transmit lower spatial-frequency flicker and motion information, are more sensitive to luminance contrast, and have higher temporal resolution. K cells are involved with the transmission of signals from shorter blue wavelengths relative to luminance [26]. The receptive fields of these cells are highly overlapping, and it is this redundancy that is responsible for the non-selective nature of SAP, with histological studies showing that a significant number of RGCs may be lost before VF deficits are manifested on SAP [27,28], overlapping RGCs covering for their lost partners. Similarly, if a specific subset of RGCs is damaged in a particular retinal region, it may not be detected as other RGC subtypes are still functioning. However, by isolating a single pathway or specific function, especially one with less redundancy, a VF deficit may be revealed even when a smaller number of cells are damaged [28]. This principle was suggested much earlier by Maddess et al. [29,30]. This rationale has led to the development of specific SAP methods described below. This technique is also termed selective automated perimetry or simply selective perimetry [31]. 

### 4.3. Short Wavelength Automated Perimetry (SWAP)

Humphrey Field Analyzer SWAP isolates short-wavelength sensitive K pathway and functions with a narrow-band blue light stimulus on a yellow illuminated background making it different from conventional SAP [32]. Comparatively, connections of the K layers are broader than that of the M and P layers. These include connections to subcortical visual centres, intracortical circuits, including V1 supragranular layers, and extrastriate cortical areas [33]. The latest version uses the SITA strategy to reduce test duration [32]. The grayscale map on SWAP reports is darker even for normal subjects because of reduced visual perception of blue cones, which should be read with caution. SWAP has higher sensitivity than full-threshold SAP in diagnosing functional deficits [34,35]. Longitudinal studies have shown that SWAP can detect VF defects 3–5 years earlier than full-threshold SAP [36,37]. SAWP is reported to be useful in DRD [38]. SWAP is limited by the yellow colour of early cataracts and acts as a blue filter causing notable diffuse reduction in sensitivity. Other disadvantages are longer test duration, patient fatigue, and discomfort [39].

### 4.4. Frequency Doubling Technology (FDT) Perimetry

In 1991, Maddess suggested the utility of frequency doubling illusion in the diagnosis of glaucoma [40]. This illusion is an apparent doubling of spatial frequency when a contrast-reversing stimulus pattern is presented at high temporal and low spatial frequency. It is suggested that this phenomenon was mediated by a subset of M cells [41]. It has also been suggested that the origin of response is cortical [42]. FDT Matrix (Carl Zeiss Meditec, Dublin, MA, USA) is an example of the technology. The targets it uses are smaller than those used in the original FDT machine, which helps 24-2 and 30-2 test patterns be congruent with SAP. Matrix provides the same reliability indices as SAP, namely fixation losses, false positives, and false negatives. The Statistical Analysis package comes with total and pattern deviation plots, glaucoma hemifield test (GHT), global indices mean deviation (MD), and pattern standard deviation (PSD). With its high sensitivity and specificity to diagnose early VF defects, FDT perimetry predicts the future onset and location of functional loss during later assessment by SAP [43]. Some prospective studies have shown that a patient with an abnormal baseline FDT report was three times more likely to develop VF defects on SAP than a patient without [44]. Other advantages of FDT perimetry are its portability, ease of execution for both operator and patient, short test duration, and resistance to optical blurring [45].

### 4.5. High-Pass Resolution Perimetry (HRP)

HRP, also known as Ring Perimetry, is a full-threshold and convenient perimetry. The test is designed for use on personal computers. It utilizes varying stimulus sizes of fixed luminance and contrast to measure threshold levels. The annular stimuli are arranged in a series of rings and have borders 5 cd/m^2^ darker and a core 5 cd/m^2^ brighter than the background. This modification effectively eliminates low spatial frequency information and creates vanishing optotypes, so the stimulus is not detectable at sizes below the acuity threshold [46]. It is suggested that HRP findings do not indicate RGC density or damage directly, even though they target the high spatial frequency parvocellular pathway [47,48]. The advantages of HRP are a shorter test duration of 5–6 min per eye, lower variability in the peripheral VF, and that it is a suitable method for the detection of early VF loss [46]. The limitations are its interference from low visual acuity and media opacities, and its large-size stimuli may limit its ability to detect small scotomas [49]. 

### 4.6. Motion Automated Perimetry (MAP)

The central few degrees of VF are important for the perception of fine detail and colour; the peripheral VF perceives motion [50]. MAP measures a coherent shift in the movement direction of a group of dots (stimuli) against a background of non-moving dots [51]. The motion size threshold is the smallest detectable circular area in which the subject can detect motion. Subjects respond by touching a computer screen with a light pen, where they detect coherent motion. Localization errors indicated as the number of pixels from the target centre, and reaction times are calculated [51]. MAP has a sensitivity of 96% and a specificity of 87% for glaucoma [52]. Long test duration of 15 min is its main limitation for its clinical utility.

### 4.7. Moorfield’s Motion Displacement Test (MDT)

The original test involved presenting a single laterally oscillating vertical-line stimulus and was shown to have higher sensitivity than SAP in detecting glaucomatous early VF defects [53]. It perceives positional displacement, providing a motion sensation. Each stimulus passes through three displacement cycles at 5 Hz [54]. The test is more resistant to the effects of cataracts compared to SAP and FDT perimetry [55]. Its advantages include being portable as the test can be conducted on a laptop computer, more robust against media opacities, high accuracy, and short test durations of 1.3 to 3.2 min per eye. MDT may provide a convenient subjective perimetric method for screening large populations [56].

### 4.8. Rarebit Perimetry (RBP)

RBP was developed by Frisen in 2002 to detect subtle VF damage [57]. Unlike other SAP, which measures the threshold for light sensitivity, RBP uses spatially and temporally placed tiny test stimuli called microdots or rare bits to avoid simultaneous stimulation of other adjacent retinal receptive fields and so improves diagnosis of the VF defects. The patient indicates whether one or two dots are observed during each presentation. RBP detects early VF defects in neurologic disorders [57,58] and glaucoma [59]. Some of the advantages of RBP are that it is simple, quick, and economical [60].

### 4.9. Microperimetry

Microperimetry, also known as fundus-controlled perimetry, incorporates static automated perimetry and a non-mydriatic fundus camera. It is directed towards testing the macula with a 10-2 test pattern or similar coarser versions. The camera tracks fundal landmarks to try to correct for any shifts in gaze and enables the manual centration of the testing grid at the anatomic fovea [61]. It was designed for concurrent observation of the fundus and correction of eye movements during perimetry [62]. It accurately locates preferred retinal location and fixation stability in subjects with low vision as reliable predictors of visual acuity estimates [63]. The scanning laser ophthalmoscope (SLO) microperimeter (SLO101) (Rodenstock, Munich, Germany) was the earliest device invented [64]. The Nidek MP-1 (Nidek Technologies, Padova, Italy) followed, which uses an LCD to present stimuli, an SLO to scan the fundus, and a fundus camera to capture images with automated real-time tracking [61,65]. OPKO/OTI (OPKO Instrumentation, Miami, FL) developed spectral optical coherence tomography (OCT) SLO, with the ability to correlate functional deficits with SLO infrared images and take bi-dimensional cross-sectional OCT images of the retina [61]. Another advantage is that SLO imaging is nonmydriatic, and the function is quite immune to changes in pupil size [66]. Nidek MP-3 comes with a wider range of stimulus intensity, from 0 to 34 decibels (dB), and has improved test–retest reproducibility [67]. CenterVue Macular Integrity Assessment (MAIA) (CenterVue, Padova, Italy) is the latest version and is quite similar to the Nidek MP-3 [61]. Microperimetry is mostly used for DRD [38]. 

## 5. Objective Perimetric Methods

### 5.1. Electroretinogram (ERG)

ERG is an electrophysiological test which records electrical response from cells within the retina as an algebraic summation of several component waves or impulses. The resting membrane potential is the mechanistic basis of impulse generation and photochemical cascade. The active electrode is placed either on the cornea or a skin electrode positioned below the margin of the lower eyelid, and a reference electrode is planted on the forehead. The potential across the two electrodes is amplified and displayed. Historically, Dewar was the first investigator to record an ERG test in humans in 1877. Most studies were performed on animals until 1941 when Riggs developed an electrode that was compatible with humans [68]. 

Full-field ERG: The standard full-field (also known as Ganzfeld, meaning whole field in German) ERG standardized by the International Society for Clinical Electrophysiology of Vision (ISCEV) has five types of recordings recorded during stimulation of the entire retina with a diffuse light [69]. It assesses generalized retinal disorders but is not suitable for detecting localized lesions. The scotopic ERG is performed after dark adaptation for 30 min. The rod responses elicited with a very dim flash of white or blue light are shown as a large b-wave and a small or non-recordable a-wave. Combined rod and cone responses are generated with a very bright white flash, providing prominent a- and b-waves. Oscillatory potentials (OPs) are obtained with bright Ganzfeld flashes. The photopic ERG is conducted after adaptation in moderately bright diffuse illumination for 10 min. To elicit cone responses, a single bright flash is given, resulting in a- and b-waves with subsequent small OPs. Flickering light stimulus at a frequency of 30 Hz stimulates cones but not the rods, providing isolated cone responses [70]. Disadvantages of full-field ERG are that the response is a total potential from different areas of the retina with different cell densities [71] and it has poor sensitivity. The ERG is normal unless more than 20% of the retina is affected [72]. 

Multifocal ERG (mfERG): To provide a form of objective perimetry, Erich Sutter utilized binary m-sequences with which it is possible to isolate electroretinogram responses from each small visual field location concurrently (hence multifocal). In fact, other sequences can be used; the main requirement is that the sequences controlling stimulus deliveries for each location be substantially uncorrelated with each other. The mfERG measures electrical activity from up to 103 retinal areas per eye within a few minutes. Enhanced spatial resolution helps the mapping and quantification of scotomas and retinal dysfunction [73,74]. The ISCEV guidelines are set out for the procedure [75]. Studies have reported that the mfERG is very sensitive to detecting VF defects in different retinal disorders such as retinitis pigmentosa [76], chloroquine- and hydroxychloroquine-induced retinal toxicity [77], age-related macular degenerations [78], glaucoma [79], birdshot retinochoroidopathy [80], and photoreceptor diseases [81]. The amplitudes were significantly smaller with longer implicit time due to immaturity of processing in the central retina [82]. Focal or foveal ERG is used to assess macular diseases. However, some studies agreed that the mfERG was not reliable in detecting local ganglion cell damage [83]. 

Pattern ERG (PERG): The PERG is generated by the RGCs and other inner retinal structures [84,85]. The stimulus used is an achromatic grating or checkerboard pattern. The PERG has a major clinical role in localizing subtle retinal pathology and optic neuropathy. For example, glaucomatous patients displayed reduced amplitudes before the scotoma was detectable [84]. The guidelines for the use of PERG are provided by the ISCEV [86]. The sensitivity of PERG to detect RGC dysfunction is increased with a high frequency of stimulus inversion presented in steady-state mode [87]. Besides its application in the diagnosis, management, and monitoring of macular, retinal, and optic nerve dysfunction, the PERG is useful in differentiating macular diseases from optic nerve diseases [88]. Macular disease is characterized by significantly reduced P50 with normal implicit time or undetectable PERG, and in optic nerve disease, the N95 amplitude is reduced, indicating primary RGC dysfunction [88]. 

### 5.2. Visual Evoked Potential (VEP)

VEP, also known as visual or visually evoked potential, visual evoked response (VER) or visually evoked cortical potential (VECP), are electrical potentials produced by a flash of visual stimulus. The recording is performed from the scalp overlying the visual cortex, and waveforms are extracted from the electroencephalogram by signal averaging. The test checks the functional integrity of the visual pathway [89]. Potentials are recorded with different arrays of electrodes, such as the 10–20 International system [90] or the Queen Square System [91]. The reference electrode is placed on the earlobe, forehead, or head midline. The ground electrode is fixed to the earlobe, mastoid process, or scalp. Pattern reversal is the most preferred stimulus as it has less inter-subject variability than flash or pattern onset stimuli. The stimuli are displayed on video monitors or CRT displays [89]. 

Multifocal VEP (mfVEP): An important development in VEPs is the introduction of the mfVEP. It isolates smaller areas of dysfunction by presenting a large number of stimulations in the same amount of time taken by the traditional methods [92]. It typically uses same binary m-sequences modulation as the mfERG [73], except that image contrast is modulated rather than luminance. The mfVEP is primarily generated in striate cortex (V1) with some contribution from the extrastriate cortex [93,94,95,96]. The common stimulus used is the dartboard or checkerboard pattern displayed on a monitor viewed at 32 cm and subtending an angle of 44.5° although there are different modifications [97]. The display is scaled cortically and consists of 64 sectors, each sector containing 16 checks, 8 black and 8 white [98]. Single-channel or the bipolar recording is done in typical mfVEP with two electrodes placed in midline over the calcarine fissure with ground electrode on the forehead or the earlobe. In multi-channel recording extra electrodes are added lateral to the inion to improve signal-to-noise ratios (SNR) [99]. The amplitudes and the waveforms are compared [100,101]. 

Pupil Perimetry: Investigators coupled infrared video to trace pupillary size with an HFA (HFA; Zeiss, Dublin, CA, USA). The software was developed to analyze pupillary responses to focal light stimuli and display results graphically. All 76 test points in the 30-2 HFA test program were tested twice to determine relative sensitivity (amplitude of pupillary constriction) and latency at each point proving pupil-based objective perimetry [102,103].

### 5.3. Multifocal Pupillographic Objective Perimetry (mfPOP)

Pupil-based multifocal testing was investigated earlier by several researchers. However, it was not appreciated because of low SNR and long test times [104]. The preliminary study in mfPOP pioneered by Maddess investigated a means of concurrently assessing VF defects of both eyes by recording pupillary responses to multifocal stimuli. They used sparsely presented stimuli to increase response reliability. The biggest advantage is that only one pupil needs to be functional to plot VFs of both eyes, and it requires no electrodes for recording, besides being an objective, non-contact, non-invasive test with a shorter test duration as both eyes are tested concurrently [104]. Responses at each tested field location are sensitivity, from the amplitude of pupillary constriction, and response delays as the time-to-peak. Response delay is an added advantage of mfPOP, which most other perimetric methods cannot provide, except mfERG and mfVEPs. Rapid coloured stimuli variants drive the parvocellular and koniocellular pathways [26,104,105] rather than the slow mid-brain pathway [106]. Amplitude is larger in the temporal than in the nasal field significantly despite the presence of the blind spot in the temporal field [49,107,108,109,110]. 

**Figure 6:**
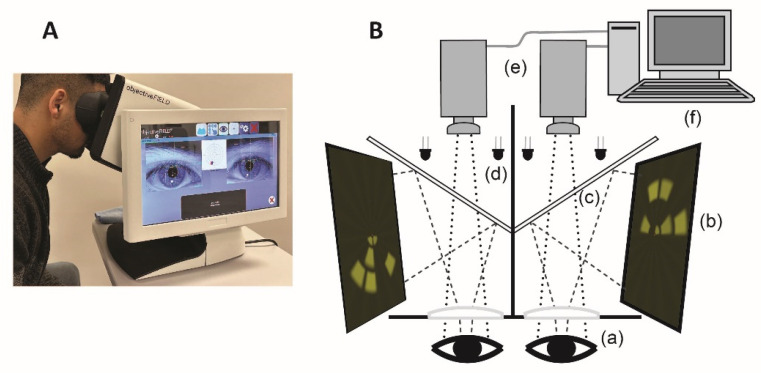
Objective perimetry. (**A**) ObjectiveFIELD Analyser (OFA), the latest version of multifocal pupillographic objective perimetry (mfPOP) developed by Konan Medical USA (Laguna Canyon Rd 150, CA 92618, United States). (**B**) Schematic diagram of mfPOP: (a): plano-convex lenses; (b): LCD monitors; (c): cold dichroic mirrors; (d): infrared emitting diodes; (e): infrared video camera; (f): personal computer. Figure 6B developed by Prof. Ted Maddess et al.

Different parts of the mfPOP are shown in Figure 6. Multiple stimuli are presented dichoptically to both eyes independently, which are reflected by cold dichroic mirrors, which transmit only infrared light. Infrared light emitting diodes (LEDs) illuminate eyes to facilitate a separate infrared video camera to capture real-time pupillary diameters, which are then extracted and retained in a computer. A pair of plano-convex lenses increases the viewing distance to optical infinity, nullifying the accommodative effects.

At a given time, stimuli are presented in different VF locations of two eyes to reduce any binocular rivalry. Both direct and consensual responses are recorded in each stimulus region. Although the inter-individual variation is high, the intra-individual responses are consistent, encouraging investigators to explore pupillography to provide objective perimetry. Pupillary responses have been elicited by various stimuli, commonly change in luminance [111,112], colour [113,114], depth perception and motion [115], and spatial content [116]. 

The objectiveFIELD Analyser (OFA), the FDA-approved version of mfPOP, is used for diagnoses and monitoring of various retinal and neurological diseases. Extensive studies have been conducted on the application of mfPOP in glaucoma [117,118]. OFA identifies changes in visual function according to the severity of diabetic retinopathy (DR) in type 2 diabetes (T2D) [119,120]. It identifies retinal functional loss before clinically detectable DR and is useful in structure–function correlation [120]. It identifies regional functional progression and recovery in T2D mild DR and DMO [121], including in children with type 1 diabetes (T1D) [122,123]. It has recently been shown to have superior diagnostic power compared to SITA_SWAP or Matrix 24-2 perimetry [124]. OFA is sensitive in detecting age-related macular degeneration (AMD) [119,125,126], multiple sclerosis [127], and epilepsy [128]. 

## 6. Discussion

Every perimetric method, qualitative or quantitative, has its own merits and demerits. Qualitative tests such as the Amsler grid may not provide a detailed exposition of lesions, but it is portable, simple, quick, and allows for home monitoring by patients [129]. It may be an ideal VF test method in regional health centres with limited resources. It relies heavily on subjective interpretation and may also be compromised by completion phenomena, which perceptually fill small gaps in line stimuli [129,130]. The tangent screen is more sensitive in delineating the borders of scotomas and is more reproducible than Amsler’s grid [130]. 

The placement of electrodes in the midline is essential while performing mfVEPs to produce identical recordings from normal subjects. However, small discrepancies of about 4 to 5 ms are found normally across the midline due to the location of the calcarine fissure relative to external electrodes and the differences in the local folding of the primary visual cortex, posing difficulty in labelling normal or diseased recordings [100,101,119]. Like the mfERG, there are no normative data for these methods; this is due to different recording methods being used by different groups. This limits mainstream clinical use. Another factor for VEPs generally is that due to the folding of Visual Area 1 around the calcarine sulcus and different brain morphology between subjects’ scalps, recorded waveform shapes can be quite variable [119,131].

SAP has been the main perimetric method for decades in clinical practices, but these, too, have limitations. Elderly people not only find difficulty in understanding and performing subjective perimetric methods but also find it stressful. In a study conducted on the performance of gold standard perimeter, HFA SITA full threshold, the specificity in normal subjects was only 38% at the first test and 73.7% after two tests [132]. So, there is a learning curve associated with subjective perimetry that complicates the interpretation in new patients, such that two to three VF tests need to be performed before a reliable result is achieved. Therefore, any objective functional information is useful, particularly among those who are poor performers in subjective perimetry [133]. SAP also has huge test–retest variability partly due to under-sampling caused by the relative influence of fixation and variation in sensitivity across a VF faster than the Nyquist for a standard sampling interval of 6°. High test–retest variability interferes with ability to track VF defect progression and is common to all variants of SAP [134,135,136]. Another limitation is that different perimeters produce quite nonlinearly related data [137,138,139]. SAP VF contains a mixture of locations with normal values, non-seeing locations, and locations with mildly or moderately reduced sensitivity values ranging from 0 to 43 dB. Matrix is provided with only 15 discrete values unevenly distributed between 0 and 38 dB, thus creating an uneven distribution of values [138]. SAP is also limited by the principle of redundancy—the receptive fields of RGCs are highly overlapping, and it is this redundancy that is considered responsible for the non-selective nature of SAP, with histological studies showing that a significant number of RGCs may be lost before VF deficits are manifested on SAP [27,28]. 

Static perimetry, the most commonly used SAP, has lower area coverage by stimuli than kinetic perimetry. Other limitations include the need for greater patient concentration, decreased efficacy in delineating complex lesions that extend into the peripheral VF and localizing lesions within the occipital lobes [140,141]. 

Objective perimetry based on pupillography, such as OFA, is gaining popularity. OFA is an objective, non-invasive, non-contact, functional perimetric method evaluating both foveal and extra-foveal function. Newer versions of OFA have higher SNR, shorter test duration and high diagnostic accuracy [38,142]. A head-to-head study validated the cortical input of OFA using mfVEP and found comparable [119]. SAP and OFA show similar structural and functional relationships with regard to retinal nerve fibre layer thickness and VF changes [143].

Conventional and rapid OFA tests have performed well in diagnosing DR and functional loss even prior to the classical DR in both adults with T2D and children living with T1D [122,123]. It is sensitive in detecting AMD [119,125,126,144] and identifies anti-VEGF-induced changes in the retinal function for exudative AMD [125,145]. There was a significant reduction in amplitudes and significant response delay (*p* < 0.001), proving OFA is a well-tolerated objective method in the diagnosis and assessment of multiple sclerosis [117,127,146]. Amplitude was found to be lower among the patients with migraines than in normal controls but did not reach statistical significance, and treatment of migraine with Triptan improved amplitudes [117]. 

Recently, much more emphasis has been given to developing OFA as a user- and patient-friendly diagnostic device. The rapid test protocols, M18 and W20, are developed, which can test both eyes with high accuracy in less than 90 s [127,142]. M18 and W20 test 18 and 20 regions/eye extending to ±10° and ±30°of VF, respectively. The sensitivity and specificity of these rapid tests were compared head-to-head with the conventional mfPOP tests taking 7 min and were reported as comparable [142]. More importantly, the M18 test has stimulus regions matching the 9 subfields of the early treatment diabetic retinopathy study (ETDRS) grid used to report macular thickness on OCT, allowing for easy functional (OFA sensitivity and delay measures) and structural (OCT thickness) data [142]. Short test duration and easy structure-function correlation make the application of these tests ideal for use among children and young people living with T1D. It is recommended that all diagnostic tests have standardized effect sizes of >2, and thus an Area under the Receiver Operating Characteristic Curve (AUROC) of >0.92, providing good diagnostic power [147]. However, relatively few tests meet this criterion. Comparatively, OFA performs better and meets these criteria [38]. Additionally, clinical guidelines are provided for the use of OFA in different scenarios such as after mydrisis, which is commonly practised in eye clinics, in particular the retinal clinics [110].

## 7. Conclusions

The selection of perimetric methods should be personalized, depending on the ability and reliability of individual patients, available resources, and earlier VF tests that have been performed. Subjective methods may not be ideal to rely on unless the patients are experienced, and so there are no further learning effects. It may be prudent to follow up and prognosticate a patient with the same perimetric method or at least one having comparable report systems. An objective, non-contact, highly reproducible, and reliable diagnostic test that takes less than 90 s to test both eyes and with easy structure–function correlation will be a game changer in busy clinics for providing standard eye care services. 

## 8. Key Messages

1. Visual field testing provides critical information on eye, brain and neurological diseases and is an integral part of comprehensive ophthalmic evaluation.
2. Subjective visual field tests, including standard automated perimetry, are limited by high test–retest variability, learning effects, variability due to under-sampling, and the principle of redundancy.
3. Among the objective tests, electroretinograms and visually evoked potentials are limited by the inconvenience of applying electrodes and are time-consuming.
4. Multifocal Pupillographic Objective Perimetry is an objective and reliable method which can test both eyes in less than 90 s and has the critical advantage of measuring response delay, which no other perimetric method provides. It has normative data.
5. Some Multifocal Pupillographic Objective Perimetry stimulus regions are matched spatially to Early Treatment Diabetic Retinopathy Study 9 subfields for easy structural-functional correlation and recommended diagnostic effect size of >2, and thus Area under the Receiver Operating Characteristic Curve of >0.92 are better met by this method than any others.

## 9. Future Directions

Current management of retinal diseases is directed to late-stage diseases such as clinically evident DR and neovascular AMD. The immediate future of disease management should target earlier-stage diseases such as functional DR and early or intermediate AMD before the emergence of classical clinical stages. Therefore, diagnostic tests must have high sensitivity and specificity to detect both disease-specific structural and functional changes. Secondly, it is preferable to have diagnostic tools with short test times and high reliability and reproducibility, which saves time for high-quality healthcare services. Perimetric methods of the future should be able to diagnose early subtle functional changes while providing personalized or targeted early treatment. These should be able to monitor the earliest changes to guide clinical trials and treatment of early stage diseases.
